# Mapping the Mechanical and Immunological Profiles of Polymeric Microneedles to Enable Vaccine and Immunotherapy Applications

**DOI:** 10.3389/fimmu.2022.843355

**Published:** 2022-03-14

**Authors:** Shrey A. Shah, Robert S. Oakes, Senta M. Kapnick, Christopher M. Jewell

**Affiliations:** ^1^ Fischell Department of Bioengineering, University of Maryland, College Park, MD, United States; ^2^ United States Department of Veterans Affairs, Vetrans Affair (VA) Maryland Health Care System, Baltimore, MD, United States; ^3^ Robert E. Fischell Institute for Biomedical Devices, University of Maryland, College Park, MD, United States

**Keywords:** microneedles, immunomodulation, nanotechnology, biomaterials, vaccines, intrinsic immunogenicity

## Abstract

Biomaterials hold great promise for vaccines and immunotherapy. One emerging biomaterials technology is microneedle (MNs) delivery. MNs are arrays of micrometer-sized needles that are painless and efficiently deliver cargo to the specialized immunological niche of the skin. MNs typically do not require cold storage and eliminate medical sharps. Nearly all materials exhibit intrinsic properties that can bias immune responses toward either pro-immune or inhibitory effects. Thus, because MNs are fabricated from degradable polymers to enable cargo loading and release, understanding the immunological profiles of these matrices is essential to enable new MN vaccines and immunotherapies. Additionally, understanding the mechanical properties is important because MNs must penetrate the skin and conform to a variety of skin or tissue geometries. Here we fabricated MNs from important polymer classes – including extracellular matrix biopolymers, naturally-derived polymers, and synthetic polymers – with both high- and low-molecular-weights (MW). We then characterized the mechanical properties and intrinsic immunological properties of these designs. The library of polymer MNs exhibited diverse mechanical properties, while causing only modest changes in innate signaling and antigen-specific T cell proliferation. These data help inform the selection of MN substrates based on the mechanical and immunological requirements needed for a specific vaccine or immunotherapy application.

## 1 Introduction

Existing pathogens and diseases continue to create challenges for current vaccine and immunotherapy technologies. The challenges span not only efficacy and selectivity, but also distribution, storage, and compliance (1, 2). Most recently, for example, the COVID-19 pandemic highlights the need for vaccines that can be easily disseminated without the need for refrigeration or complex cold chains (3). Also evident is the need for vaccines that generate potent and durable responses (4). Likewise, in cancer immunotherapy – where the target antigens are on cancerous host cells or tissues, there is a great need for safe and selective approaches that generate strong responses against difficult-to-detect tumor antigens. Integrating new engineering technologies to improve the distribution, storage, and performance characteristics of vaccines and immunotherapies could enable next-generation vaccines that are easily deployed and generate strong and selective outcomes.

Biomaterials – including polymer and lipid nanoparticles, engineered scaffolds, and biodegradable materials – are being intensely investigated for vaccines and immunotherapies across infectious disease (5, 6), cancer (7–9), and autoimmunity (10–12). These materials can be either naturally occurring, fully synthetic, or hybrid in composition. Across these categories, a generally attractive feature is the ability for improved levels of control. For example, many biomaterials provide tunable control over loading and release of multiple immune cues, targeting specific cells or tissues, cargo protection, and control over release kinetics (13). The ability to chemically modify and functionalize the surface of nano- or microparticles also offers a modular ability that is particularly attractive in displaying immune cues to direct immune processes (14–16).

As alluded to earlier, one key ability of biomaterials is targeting specific tissue niches. In this context, one emerging approach particularly relevant for vaccines and immunotherapies are microneedles (MNs). MNs are small arrays of micrometer-sized needles made of synthetic or natural matrices. The design and length scale of these technologies ensure delivery of cargo across the skin barrier, and efficient access to the unique immunological niche within the skin (17). Because the skin is immunologically rich in specialized antigen presenting cells (APCs) – such as dendritic cells (DCs) and Langerhans cells (LCs), this organ is an important target for vaccine and immunotherapy (18). One design requirement for MNs is lengths sufficient to penetrate the skin, typically in the range of 25-500µm.

Beyond skin targeting, MNs provide other advantages including painless delivery – since the needles are too short to reach pain receptors, elimination of medical sharps, and incorporation and release of multiple immune cargos. Importantly, because the synthetic or natural polymer matrices used to synthesize MNs typically stabilize biological cargo, MNs often eliminate the need for refrigeration or cold-chain distribution (19). MNs can be either solid, coated, degradable, or hollow, depending on the application. Further, they can exhibit a range of mechanical properties – stiffness or flexibility, for example - that can determine the applications and features (e.g., skin penetration). Further, biomaterials can exhibit intrinsic immune profiles that can be immune activating or even anti-inflammatory (14, 20, 21). Thus, as with all biomaterials, the potential of MNs also requires additional attention to understanding how these matrices might interact with skin and immune cells, along with the other vaccine or immunotherapy components.

Toward this need, here we focused on understanding the mechanical and immunological profiles of key classes of polymer matrices used to form degradable MNs. Degradable or dissolvable designs are particularly relevant for immune applications because this strategy enables the encapsulation of cargo and degradation or dissolution to deliver cargo with controlled-release kinetics (22–24). Some of the key degradable polymers employed for MNs include gelatin, carboxymethyl cellulose (CMC), polyvinyl alcohol (PVA), polyvinyl pyrrolidone (PVP), and hyaluronic acid (HA) (25–31). Of note, gelatin-based MNs recently cleared phase I clinical trials for influenza (32). For the current studies, we selected six degradable polymer matrices from both natural and synthetic origins, testing high and low molecular weight (MW) formulations for each matrix type. The immunomodulatory properties of these MNs were characterized using DC activation studies, T cell co-cultures, and gene expression studies. In parallel, key mechanical characteristics of these degradable - polymer MNs including fracture force and stiffness - were characterized to assess the ability of MNs to penetrate the skin and conform to different locations and organ geometries (i.e., stiffness). These studies revealed the MN matrices had diverse mechanical properties and caused modest – though statistically significant – changes to immune signaling as a result of intrinsic immune profiles. These studies contribute to strategies for selecting MN matrices appropriate for specific immune engineering applications with respect to both mechanical and immunological performance characteristics.

## 2 Materials and Methods

### 2.1 Microneedle Matrices Used for the Experiments

Low Bloom and High Bloom Gelatin, Sodium Carboxymethyl Cellulose (90kDa and 700kDa), Dextran (9-11kDa and 150kDa), PVA (13-23kDa and 85-124kDa), and PVP (10kDa and 1300kDa) were purchased from Sigma Aldrich, U.S.A. HA (<10kDa and 100-150kDa) was purchased from Lifecore Biomedical, USA.

### 2.2 Fabrication of MNs

MNs were fabricated using a solvent casting process using an MN master and polydimethylsiloxane (PDMS) (SYLGARD Kits, DOW, 184 SIL ELAST KIT) molds. This method was adapted with modifications from our lab’s previous work on MNs (33), including careful considerations to the purity and avoiding endotoxin. A 5% w/w of the polymer solution was pipetted into the PDMS mold. The PDMS mold was then centrifuged at 4000g for 10min to fill the tines. This centrifuged PDMS mold was dried for 24-48h followed by releasing carefully to obtain the degradable polymer MNs.

### 2.3 Scanning Electron Microscopy

All MNs were imaged using a Phenom XL G2 Desktop SEM at 180x magnification, high vacuum, accelerating voltage of 15kV, and using a backscattered electron detector (BSD). To get a complete picture of the tines, the MNs were mounted on a 45° stud.

### 2.4 Mechanical Properties

To characterize the mechanical properties (stiffness and fracture force) of the MNs, a Dynamic Mechanical Analyzer (DMA) TA Q800 was used in the static stress-controlled mode using a compression clamp. In brief, the MN array was fixed on the lower plate, and the upper plate was moved towards the MN array in the strain ramp mode of the instrument at the speed of 0.01mm/s. The compression was stopped once the loading displacement reached 300µm. For approximating the stiffness of the MNs, blocks of polymers (cuboidal shapes 1cm x 1cm x ~3mm) were used. Qualitatively, stiffness was also measured by compressing the MNs using a tweezer and taking images before and after compression.

### 2.5 Characterizing Immunomodulatory Properties

#### 2.5.1 DC Activation Studies

For DC Activation studies, primary DCs were isolated from spleens of naïve C57BL/6 mice using CD11c+ magnetic isolation beads (Miltenyi, 130-108-338). Spleens were isolated, minced, and incubated in Spleen Dissociation Media (StemCell Technologies, 07915), dissociated using a 16G needle, passed through a 40μm strainer, resuspended in MACS buffer containing CD11c+ magnetic isolation beads, and passed through an LS column in a magnet, with CD11c+ cells being collected in a final wash. Isolated DCs were plated at a density of 100,000 cells per 200μL in wells of a 96-well plate. These CD11c+ DCs were stimulated with MN tines dissolved in DC Media. Cells treated with LPS (1µg/mL), and cells treated with PBS were used as a positive and negative control, respectively. Polymer concentrations ranging from 0.0001-100µg per well for each lower and higher MW polymer were used for the treatment groups. After incubation with the groups for 24h, the DCs were washed twice with FACS buffer and then blocked using Fc Block (25X dilution, BD biosciences) for 10min at room temperature. The cells were then stained with antibodies for CD80, CD86, CD40, and viability. All antibodies were fluorescent conjugates and were used by staining for 20min at a 1:100 dilution in FACS buffer (for CD80, CD40, and CD86) and 1:200 dilution for Viability dye. Cells were then washed twice with FACS buffer for analysis by flow cytometry. Flow cytometry was performed on a FACS Celesta (BD Biosciences) and CytoFLEX flow cytometer (Beckman Coulter), and analyzed using FlowJo. For gene expression analysis, DCs were cultured for 24h with LPS and MN substrate or MN substrates alone prior to isolation and analysis.

#### 2.5.2 T Cell Co-Culture

To see the effect of the MN matrices on T cell proliferation and expansion, CD11c+ DCs isolated as previously mentioned were treated with the MN solution (0.0001-100µg), LPS, and PBS. Soluble SIINFEKL (5µg/mL) was also pulsed into the wells along with the MN substrates and controls. After 48h, T cells isolated from OT-1 mice using CD8+ T cell negative selection kits (StemCell Technologies, 19852) were stained with cell proliferation dye eFluor 670 (0.5µM/well) during a 5 min incubation at room temperature. T cells were then co-cultured with each DC sample by adding 3x 10^5^ T cells per well (making the ratio 1:3 DC to T cells). T cell proliferation was determined by the mean fluorescence intensity of the eFluor 670 signal and compared with the positive and negative controls.

#### 2.5.3 Gene Expression Analysis

For gene expression analysis, RNA was isolated using the Quick-RNA Microprep Kit (Zymo Research, R1050), where cells were lysed in their wells using a lysis buffer, genomic material was captured in a silica-based matrix, and DNA was degraded with DNase I. RNA was diluted to 20ng/μL in RT-qPCR grade water (Thermo Fisher, AM9935). cDNA was reverse transcribed using the High-Capacity cDNA Reverse Transcription Kit (Thermo Fisher, 4368813). The qPCR reaction mix was made using TaqMan Gene Expression Assay probes in TaqMan Gene Expression Master Mix (Thermo Fisher, 4369016). Taqman probes utilized were: glyceraldehyde 3-phosphate dehydrogenase (*Gapdh*), Mm99999915_g1; actin beta (*Actb*), Mm00607939_s1; 18s rRNA (*18s*), Mm00434228_m1; interleukin 6 (*Il-6*), Mm00446190_m1; interleukin 10 (*Il-10*), Mm99999067_m1; interferon-gamma (*Ifn-γ*), and Mm00441891_m1; tumor necrosis factor (*Tnf-α*). qPCR was performed in a MicroAmp Optical 384-well reaction plate (Applied Biosystems, 4309849) with optical adhesive film (Applied Biosystems, 4360954) on a QuantStudio 7 Flex Real-Time PCR System (Applied Biosystems, 4485701).

### 2.6 Animal Care

All animal care and experiments were carried out in compliance with federal, state, and local guidelines and using protocols reviewed and approved by the University of Maryland’s Institutional Animal Care and Use Committee (IACUC).

### 2.7 Statistical Analysis

All characterization studies were replicated at least three times, and all data points, along with mean ± standard deviation, were reported. Cellular analyses were replicated at least twice to ensure reproducibility of biological effects. For DC activation, T cell studies, and RT-qPCR studies, one-way ANOVA with Tukey post-test corrections for multiple comparisons were used to compare groups. Analysis and hierarchal clustering were done in MATLAB v.R2019b using the clustergram function, where the data were standardized for each gene to compare across multiple groups, and clustering was performed using a single linkage (nearest neighbor). Statistical calculations were performed using GraphPad Prism v.9.1.0.

## 3 Results

**Figure 1:**
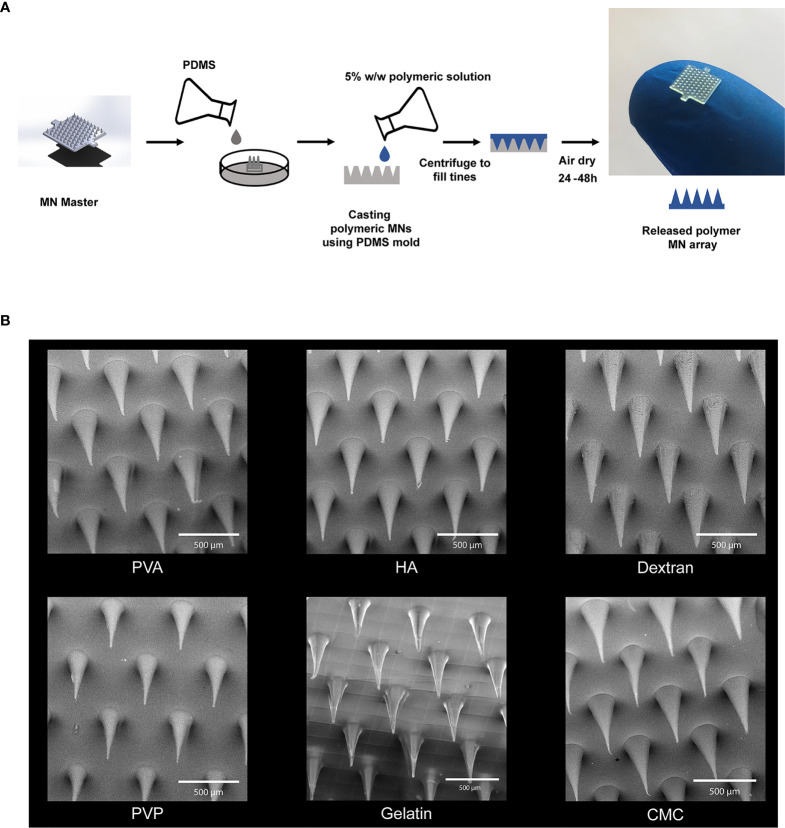
Microneedles were fabricated using six different polymers. **(A)** Schematic of fabrication scheme for making the MNs. **(B)** SEM Images of the MNs fabricated using high MW degradable polymers. Scale bar = 500µm.

We began by assessing polymers with three different origins 1) those derived from the extracellular matrix - gelatin and hyaluronic acid (HA), 2) naturally-derived polymers - carboxymethyl cellulose (CMC) and dextran, and 3) synthetic polymers - polyvinyl alcohol (PVA) and polyvinylpyrrolidone (PVP). MNs were prepared from each material class by a solvent casting fabrication method involving a MN master and PDMS mold (**Figure 1A**). This process allowed facile manufacturing of MNs irrespective of matrix type. Scanning electron microscopy (SEM) revealed well-defined geometries that maintained the fidelity of the PDMS molds (**Figure 1B**). These data also confirmed the expected length scale for each matrix, with MNs exhibiting lengths of 500µm-520µm and base diameters of 200µm-220µm.

**Figure 2:**
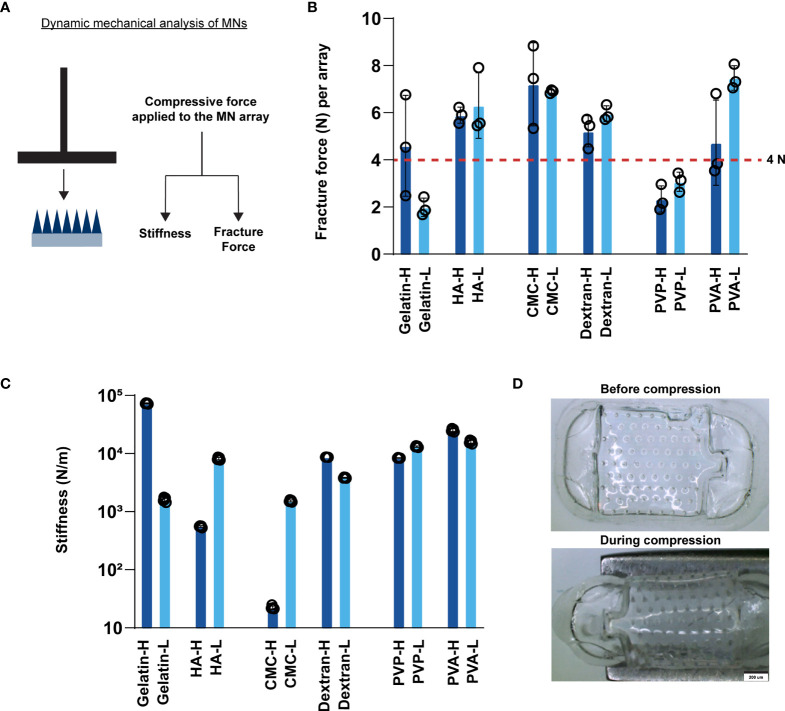
MNs exhibit varying stiffness and fracture forces as a function of matrix type. **(A)** Schematic showing the experimental setup for measuring fracture force and stiffness. **(B)** Fracture force of the fabricated MN arrays. The red horizontal line at 4N indicates the minimum force required to penetrate the skin per array. **(C)** Stiffness of the fabricated MNs. **(D)** Images of high MW CMC MNs before and during compression.

Next, we assessed important mechanical properties of MNs using dynamic mechanical analysis, including fracture force, the force required for mechanical fracture of the MNs (34). This parameter determines if a particular MN can support the force needed to penetrate the skin. Additionally, we assessed stiffness, the extent to which an object resists deformation in response to an applied force. Thus, stiffness determines if MNs are stiff enough to support higher pressure contact with skin– such as during a transient application with a quick dissolution design, or flexible enough to be applied to non-flat geometries with conformal contact, such as a slow release application requiring skin contact for longer durations (e.g., hours, days). We measured fracture force and stiffness using compressive forces applied during DMA (**Figure 2A**). Fracture force and stiffness studies revealed dramatic differences in the properties of MNs formed in this library, both as a function of polymer structure, and in some cases, also as a function of MW (e.g., CMC) (**Figures 2B–D**). Notably, most matrices exhibited fracture forces greater than 4N (**Figure 2B**), the minimum force required to penetrate the skin for these geometries (24). In contrast, gelatin (low MW) and PVP MNs exhibited fracture forces < 4N (**Figure 2B** and **Supplementary Table 1**), suggesting these designs might fracture before penetration. The stiffness varied over several orders of magnitude, an important finding since different applications may require MN patches that are either rigid or flexible (**Figure 2C** and **Supplementary Table 2**). Interestingly, some of the matrices afforded high fracture forces at both MWs but allowed stiffness to be controlled to achieve either flexible or rigid materials that both support skin penetration (e.g., CMC). **Figure 2D** demonstrates the flexibility of CMC MN arrays using images obtained before and during compression. In general, lower stiffness values corresponded to flexible MNs. This flexibility, while maintaining sufficient fracture force limits on the actual needles, could be useful when applying MNs to geometries or skin that are not flat, facilitating contact and adhesion (35, 36).

**Figure 3:**
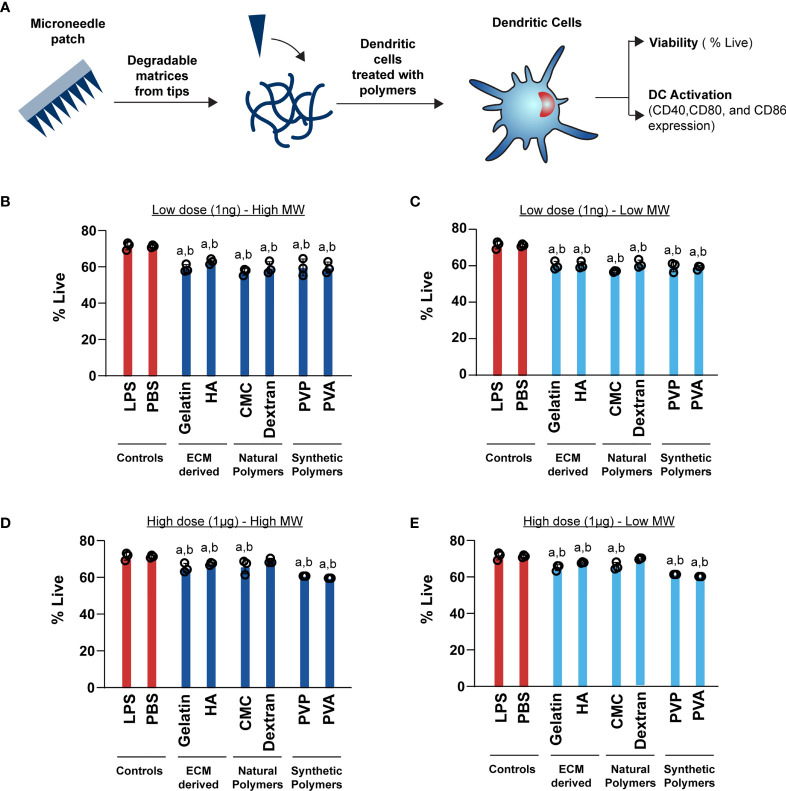
High (1μg) and Low (1ng) doses of both high and low MW polymers have a moderate effect on viability. **(A)** Schematic showing the experimental setup for DC activation **B**) Viability of cells treated with 1ng of high MW polymers **(C)** Viability of cells treated with 1ng of low MW polymers **(D)** Viability of cells treated with 1μg of high MW polymers **(E)** Viability of cells treated with 1μg of low MW polymers. For panels **(B–D)**, “a” and “b” represents significant statistical differences (p < 0.05) when compared with LPS and PBS group, respectively.

After testing the mechanical properties of the MNs, we carried out a series of studies to characterize the intrinsic immune profiles of the MN matrices. We began these studies with viability assessment in primary mouse DCs (**Figure 3A**). All of the MN matrices generally afforded good viability for both high MW (**Figures 3B, C**) and low MW (**Figures 3D, E**) designs. Relative to positive and negative controls, there were statistically significant decreases, though these were modest at 5-9%. We also varied the dose to determine how sensitive DCs might be to the mass of MN matrix encountered during the application of MNs loaded with immune signals. However, viability was not significantly impacted as a function of dose across three orders of magnitude (i.e., 1ng *vs*. 1μg) (**Figures 3B** *vs*. **3D**; **Figures 3C** *vs*. **3E**).

**Figure 4:**
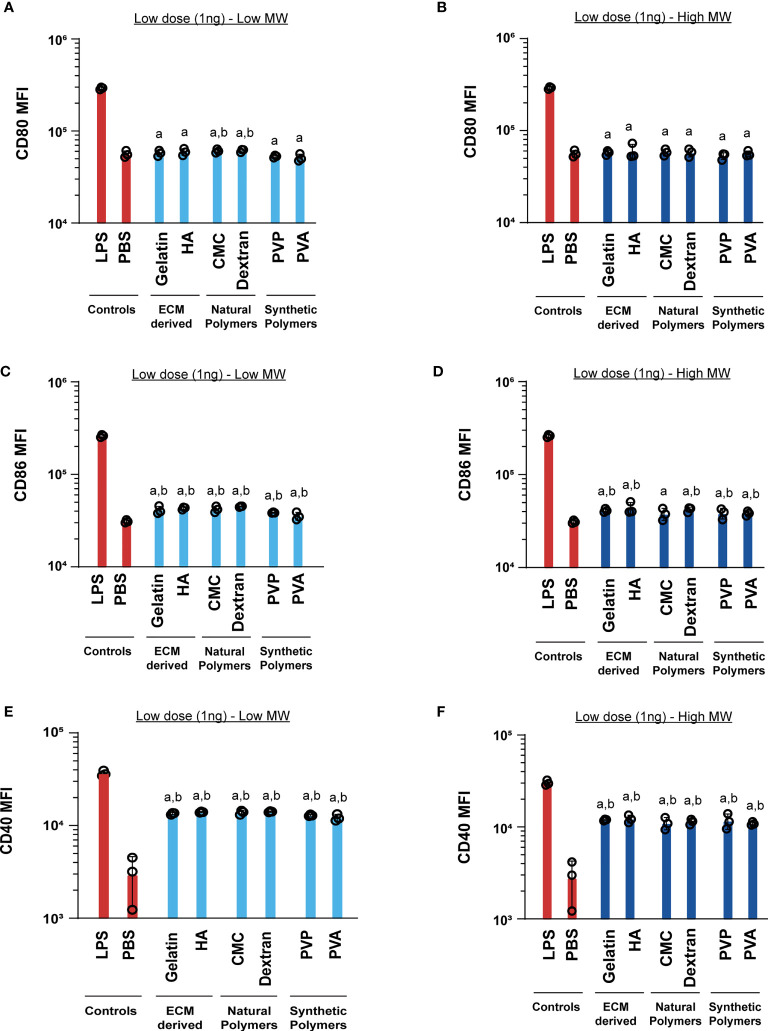
Low (1ng) doses of both high and low MW polymers lead to low activation levels. Activation of DCs treated with low or high MW polymers at low doses, respectively, as indicated by expression of **(A, B)** CD80, **(C, D)** CD86 and **(E, F)** CD40. “a” and “b” represents significant statistical differences (p < 0.05) when compared with LPS and PBS group respectively.

**Figure 5:**
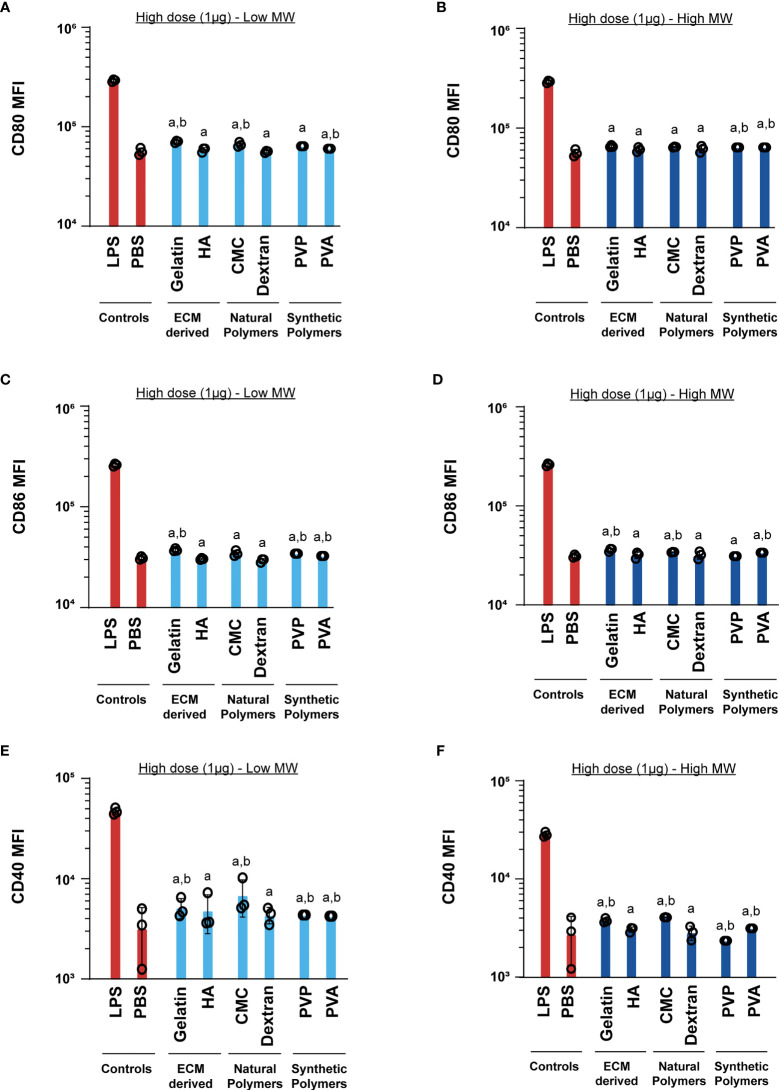
High (1μg) dose of both high and low MW polymers leads to activation similar to the negative control. Activation of DCs treated with low or high MW polymers at high doses, respectively, as indicated by expression of **(A, B)** CD80, **(C, D)** CD86 and **(E, F)** CD40. “a” and “b” represents significant statistical differences (p < 0.05) when compared with LPS and PBS group respectively.

Next, we tested if these MN designs alter DC activation profiles. These studies were initially conducted by incubating cells with the low dose (1ng) of MN matrices. Generally we found that the matrices did not increase CD80 relative to PBS, for either low or high MW polymers (**Figures 4A, B** **)**. One exception was a modest increase observed during treatment with low MW versions of the natural polymers, CMC and dextran. For CD86, nearly all polymers led to a small, but statistically significant increase in activation relative to PBS (**Figures 4C, D** **)**. In contrast, CD40 expression was significantly higher for all the MN matrices when compared to the PBS group (**Figures 4E, F** **)**. However, in all cases the LPS positive control led to much higher activation than relative to the MN matrices (**Figure 4**). This indicates that at low doses, the MN substrates have some immune-activating properties, which are significantly less than LPS. Next, we tested a higher dose of the MN matrices (1µg) with DCs. Similar to the low dose treatment, most MN matrices either did not increase activation of CD80, CD86, and CD40, or led to very small increases (**Figure 5**). Interestingly, although the MN matrices slightly increased CD40 expression at the higher dose (**Figures 5E, F** **)**, the magnitude was less than that observed for the low dose treatments (**Figures 4E, F** **)**. This may indicate engagement of feedback mechanisms during encounter of high or persistent polymer doses (37, 38). However, for all doses and all polymers, activation was modest relative to LPS. Even so, the variation in expression reveals the unique immune-activating properties that vary as a function of both composition and dose; the latter is driven by proximity of cells to the MN insertion site and the diffusion or clearance of degradation byproducts.

**Figure 6:**
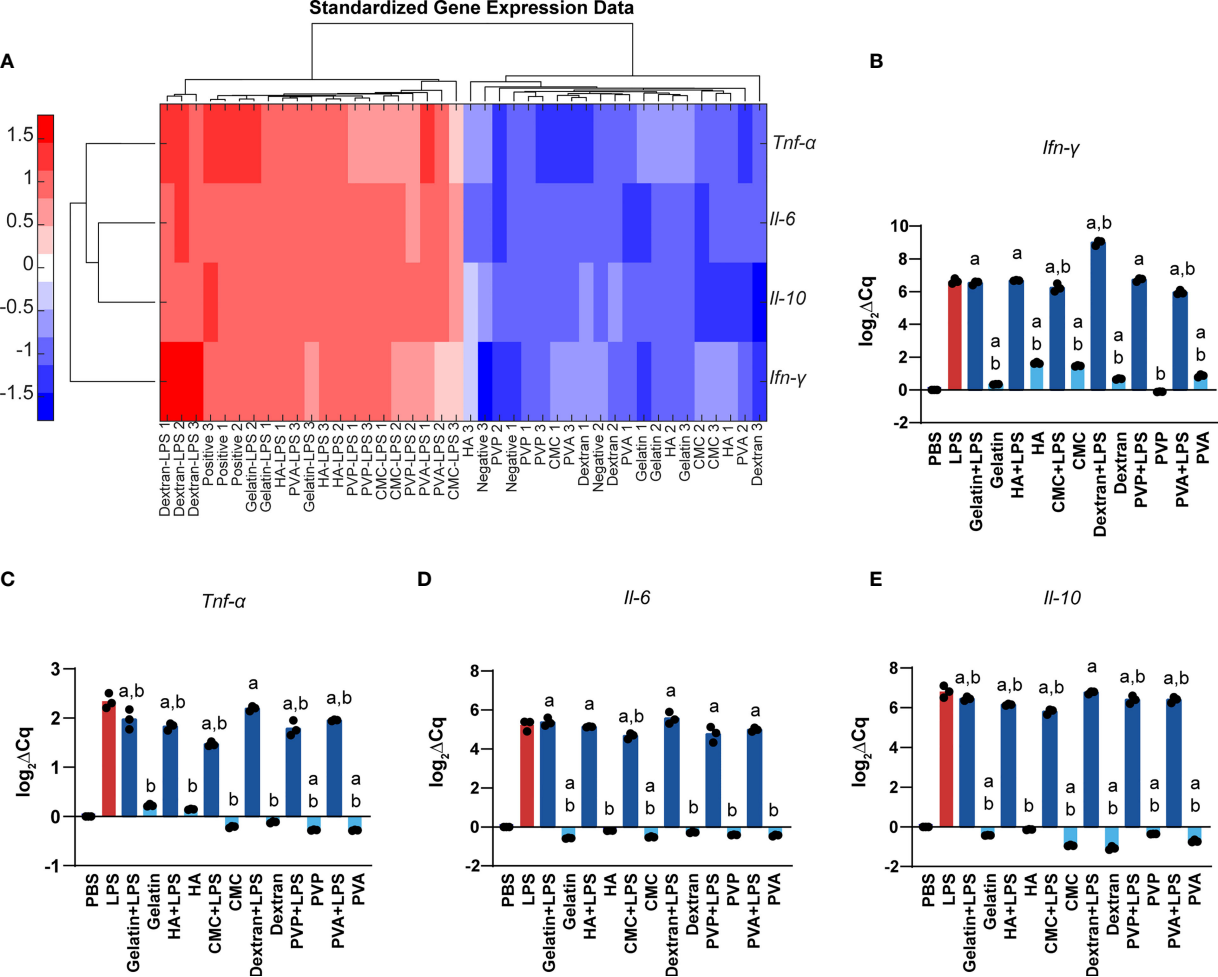
Polymer MNs have significant but modest differences in gene expression of inflammatory as well as regulatory cytokines. **(A)** Heat map and log2 gene expression data for **(B)** *Ifn-γ*, **(C)** *Tnf-α*, **(D)** *Il-6*, and **(E)** *Il-10*. “a” and “b” represent significant statistical differences (p < 0.05) when compared with PBS and LPS group respectively.

We next used RT-qPCR to measure gene expression changes in DCs as a function of MN matrix type and MW. To assess the intrinsic immune profiles of these materials, we measured the gene expression after 24h of cell incubation with MN matrices. We selected genes *Tnf-α, Ifn-γ*, and *Il-6*, which are common innate inflammatory cytokines, and *Il-10*, a common regulatory cytokine. As expected, stimulation with LPS significantly increased the expression of *Tnf-α, Ifn-γ, Il-6*, and *Il-10* when compared to treatment with PBS or MNs matrices. This was evident in unsupervised clustering of the gene expression heat map (**Figure 6A**). In assessing *Ifn-γ*, we discovered a modest activating effect relative to PBS - except for PVP, consistent with the prior studies using polymers in soluble non-MN form that natural and synthetic matrices activate innate inflammatory pathways (**Figure 6B**). When LPS was also present, the addition of MN matrices generally did not further increase expression of *Ifn-γ*, though one notable exception was dextran (**Figure 6B**). For *Tnf-α*, MN matrices alone did not meaningfully alter gene expression compared to PBS (**Figure 6C**). However, with LPS also present, there was a small but significant decrease in *Tnf-α* expression relative to LPS alone for most of the MN matrices (**Figure 6C**). MN treatment caused only very small perturbations in *Il-6* gene expression, with or without the presence of LPS (**Figure 6D**). Likewise, for *Il-10*, we saw some statistically significant decreases for MN matrices in both the absence and presence of LPS, though these were also modest in magnitude (**Figure 6E**). Taken together, this gene expression analysis is consistent with the DC activation studies, suggesting the MN matrices have intrinsic immune properties that modestly impact signaling and activation, despite creating opportunities for vastly different mechanical properties to support specific design applications.

**Figure 7:**
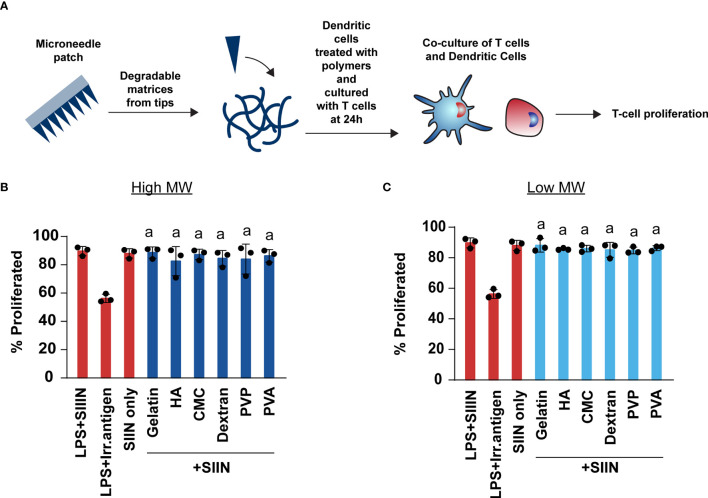
Polymer MNs do not limit the ability of T cells to engage with their antigen or proliferate. **(A)** Schematic showing experimental set-up for T cell co-culture experiments. **(B, C)** Proliferation of T cells when treated with high and low MW polymer MNs, respectively. “a” represents significant statistical differences (p < 0.05) when compared with LPS+ irrelevant antigen (irr. antigen). The comparison between LPS+SIIN and SIIN with the polymer MNs was not significant.

We next investigated whether MNs enhance or inhibit the ability of T cells to engage their cognate antigen and proliferate. These studies were carried out by treating DCs with MN matrices, adding in a model antigen – SIINFEKL, then co-culturing these DCs with T cells from OT-I transgenic mice. OT-I T cells are specific for SIINFEKL, causing proliferation and activation when T cells encounter SIINFEKL displayed in MHC-I by DCs (**Figure 7A**). We choose to use the high dose (1μg) as this dose is representative to the cargo: matrix ratio used in immune engineering applications. In all cases, we observed T cell proliferation was unaffected by the presence of MN matrices when compared to SIIN only and SIIN+LPS group (**Figures 7B, C** and **Supplementary Figure 1**). These proliferation levels were also much higher than those observed during the treatment of cells with LPS and an irrelevant antigen (Irr. Antigen). Overall, these data indicate that these MN matrices do not alter the ability of T cells to engage with a cognate antigen.

## 4 Discussion

As the applications of MNs increase there is a greater need to understand the immunomodulatory properties of the materials used to fabricate them. Here we fabricated MNs from polymers with diverse origins, assessed their suitability to penetrate the skin by measuring important mechanical properties, and characterized their intrinsic immunomodulatory properties. Creating these types of profiles is needed to enable robust MN technology platforms for a variety of applications. For example, in cancer and infectious disease, strong pro-immune outcomes are desired, whereas in tolerance or allergy applications, anti-inflammatory features may be useful. Likewise, different mechanical properties are amenable to different applications and wear-times; longer durations, for example, require increased flexibility to maintain adhesion to complex tissue or body geometries.

For the reasons just mentioned, translating MNs from bench to clinic also requires attention to fabrication processes. We used a solvent casting method for fabricating the MNs (39–41), which is more reproducible and robust compared to other techniques like microlithography and laser ablation (42). The manufacturing technique can also impact the mechanical properties, including key parameters, such as fracture force and stiffness: with insufficient fracture force values, MNs could fracture during insertion; with inappropriate levels of stiffness/flexibility the MN patch application, adhesion, and durability may be mismatched with the intended application (43, 44). We found most of the matrices tested could suitably penetrate skin, but interestingly, that some materials - such as CMC, could be prepared over a large range of flexibilities.

MNs are specialized for skin delivery, which creates unique relevance for immunoengineering applications. DCs, for example, are a major type of skin-resident immune cells involved in presenting antigen and co-stimulatory cues T cells. When T cells bind antigen and costimulatory signals on the surface of DCs, these cells differentiate and proliferate to mount immune responses against that antigen (45). This is one of the basic premises for vaccines, and also for many antigen-specific immunotherapies (46). We used splenic DCs in our experiments to facilitate the large number of primary cells needed to screen the library of matrices and doses. These cells do share important broad activating characteristics with skin-derived APCs. For example, Langerhans Cells and splenic DCs upregulate MHC, CD40, and CD80 during expression (47, 48). However, directly assessing the unique features of specific skin-resident populations will be important in future studies centered on specific immunological applications. Across the matrices tested, we generally found good viability profiles, and low levels of intrinsic inflammatory activity associated with the matrices. Although there were some increases in activation as a function of MN composition, these increases were always small relative to LPS. One interesting result was the lower activation of CD40 for high doses, compared to low MN doses. This could be due to the engagement of immunological feedback mechanisms at these higher doses. Along similar lines, in our gene expression studies, we observed a few instances where specific materials modestly dampened the activating ability of LPS. However, none of these changes limited the ability of T cells to engage their cognate antigen. Because each matrix has a slightly different immunomodulatory profile – and these profiles are generally mild in either pro- or anti-inflammatory nature - this creates opportunity for design. For example, these subtle changes might help bias responses toward a desired outcome – immunity or regulation – based on the other components in a vaccine or immunotherapy.

In summary, our studies reveal that these polymer MN substrates have profiles varying in both mechanical and immunological properties, allowing selection of MN substrates for different applications based on a combination of requirements in each of these areas. Our work is distinct from past MN studies in that we have characterized a range of matrices in these area, rather than focus on a specific vaccine candidate. This type of comparative benchmarking of intrinsic properties is important to support MN technology development and also for new vaccines and immunotherapies (49). However, understanding how the subtle changes we observed in innate signaling connect to downstream outcomes – such as T cell polarization and phenotype, is an important next step. Likewise, extending these types of studies to other skin APC types, including LCs will also be important to broaden the relevance. Lastly, a goal is to use the profiles generated in these and other studies with MN designs integrating immune signals, rather than just the matrix. This will reveal and help isolate the interplay between background intrinsic matrix effects, and the impact of the active immune cues included in emerging vaccines and immunotherapy candidates.

## Data Availability Statement

The raw data supporting the conclusions of this article will be made available by the authors, without undue reservation.

## Ethics Statement

The animal study was reviewed and approved by University of Maryland Institutional Animal Care and Use Committee.

## Author Contributions

SS, RO, SK, and CJ conceived the studies. SS and RO carried out the studies. SS, RO, and CJ carried out the data analysis. SS and CJ wrote and revised the manuscript with input from all authors. All authors contributed to the article and approved the submitted version.

## Funding

This work was supported by NIH # R01 AI144667. RO is a Career Development Awardee of the United States Department of Veterans Affairs (# IK2 BX005061).

## Conflict of Interest

CJ and RO are employees of the VA Maryland Health Care System. CJ has an equity position with Avidea Technologies. The views reported in this paper do not reflect the views of the Department of Veterans Affairs or the United States Government.

The remaining authors declare that the research was conducted in the absence of any commercial or financial relationships that could be construed as a potential conflict of interest.

## Publisher’s Note

All claims expressed in this article are solely those of the authors and do not necessarily represent those of their affiliated organizations, or those of the publisher, the editors and the reviewers. Any product that may be evaluated in this article, or claim that may be made by its manufacturer, is not guaranteed or endorsed by the publisher.
