# Supplementary material for: Mapping the Mechanical and Immunological Profiles of Polymeric Microneedles to Enable Vaccine and Immunotherapy Applications
Source: Front Immunol. 2022 Mar 14;13:843355. doi: 10.3389/fimmu.2022.843355 (PMC8964051; doi:10.3389/fimmu.2022.843355)
Supplement: Supplementary file 1 [file DataSheet_1.docx]

Mapping the mechanical and immunological profiles of polymeric microneedles to enable vaccine and immunotherapy applications

Supplementary Material

Shrey A. Shah^1^, Robert S. Oakes^1,3^, Senta M. Kapnick^1,3^, Christopher M. Jewell^1,2,3^

^1^ Fischell Department of Bioengineering, Department of Bioengineering, University of Maryland, College Park, MD, USA.

^2^ Robert E. Fischell Institute for Biomedical Devices, College Park, MD, USA.

^3^ Department of Veterans Affairs, Baltimore VA Medical Center, Baltimore, MD, USA.

*** Correspondence:**Christopher M. Jewell
cmjewell@umd.edu

**SUPPLEMENTARY FIGURES AND TABLES**

**Supplementary Table 1.** Statistical comparisons between the fracture force of different MNs. “ns” represents non-significance, “*” represents p-value<0.05, “**” represents p<0.01, “***” represents p<0.001 and “****” represents p<0.0001, respectively.

**Supplementary Table 2.** Table depicting the statistical comparisons between the stiffness of different MNs. “ns” represents non-significance, “*” represents p-value<0.05, “**” represents p<0.01, “***” represents p<0.001 and “****” represents p<0.0001, respectively.

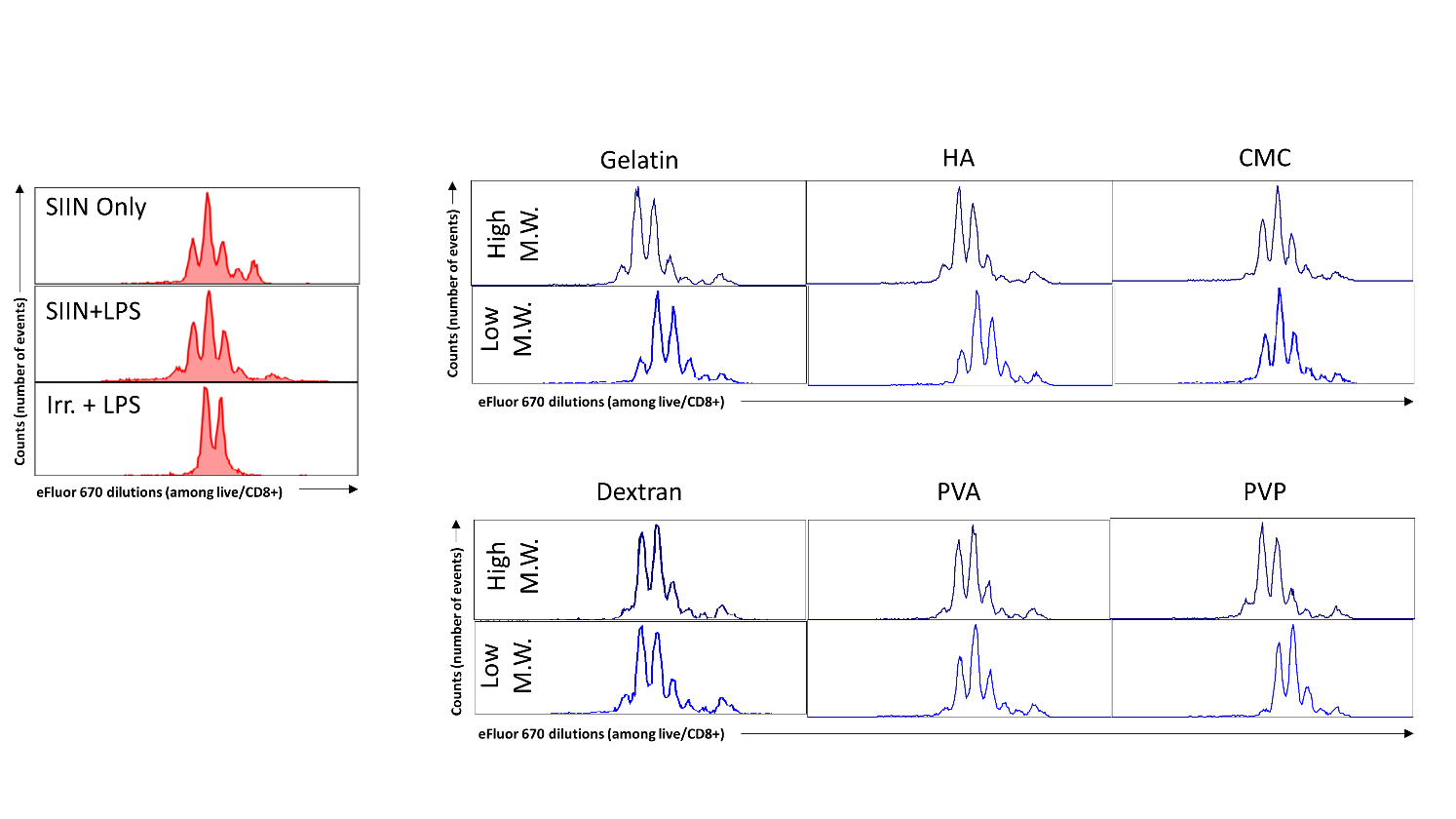


**Supplementary Figure 1.** Histogram depicting number of generations (divisions) and proliferation in OT-1 T cells labeled with eFluor 670 prior to coculture with DCs incubated with MN substrates.
